# The Kids FACE FEARS Pragmatic Randomized Trial of Therapist-Led vs Guided Internet Cognitive Behavioral Therapy for Pediatric Anxiety: Rationale and Methods

**DOI:** 10.1016/j.jaacop.2025.12.003

**Published:** 2025-12-24

**Authors:** Jonathan S. Comer, Donna B. Pincus, Molly C. Adrian, Gary McCreary, Leslie Miller, Tomas Munarriz, Kathleen Myers, Karen Xiomara Pierre-Louis, Rheanna Platt, Melissa K. Ripley, Andrea E. Spencer, Haniya Saleem Syeda, Margarita Alegría, Amelia Brandt, Carolina Costa, Lindsay Cooper, Stefany Coxe, Annie W. Dantowitz, Anthony Steven Dick, Alyssa M. Farley, Jami M. Furr, Alex E. Keller, Julia A. Lejeune, Lauren F. McLellan, Dana L. McMakin, Rachel A. Merson, Ricardo F. Muñoz, Ronald M. Rapee, Kendra L. Read, Sara Rivero-Conil, Bridget Poznanski, Michelin Jane Janvier, Hanan N. Salem, Philip Shumway, Jennifer Sikov, Michelle V. Porche, Lisa R. Fortuna

**Affiliations:** a Florida International University, Miami, Florida; b Boston University, Boston, Massachusetts; c University of Washington, Seattle, Washington; d Johns Hopkins University School of Medicine, Baltimore, Maryland; e Simmons University, Boston, Massachusetts; f Kids FACE FEARS Parent and Family Advisory Council, Chelsea, Massachusetts; g Kids FACE FEARS Parent and Family Advisory Council, Melrose, Massachusetts; h Ann & Robert H. Lurie Children’s Hospital of Chicago and the Feinberg School of Medicine–Northwestern University, Chicago, Illinois; i University of California, Los Angeles, California; j Harvard Medical School, Massachusetts General Hospital, Boston, Massachusetts; k Cedars-Sinai Medical Center, Los Angeles, California; l InStride Health, Wellesley Hills, Massachusetts; m Boston Child Study Center, Boston, Massachusetts; n University of Illinois Chicago, Chicago, Illinois; o Macquarie University, Sydney, Australia; p Palo Alto University, Palo Alto, California; q University of California, San Francisco, San Francisco, California; r Nicklaus Children’s Hospital, Miami, Florida; s Boston Medical Center, Chobanian & Avedisian School of Medicine, Boston University, Boston, Massachusetts; t University of Miami Miller School of Medicine, Miami, Florida; u University of California, Santa Barbara, Santa Barbara, California; v University of Tulsa, Tulsa, Oklahoma; w University of California Riverside School of Medicine, Riverside, California

**Keywords:** anxiety, cognitive behavioral therapy, guided iCBT, iCBT, telehealth

## Abstract

**Objective:**

Pediatric anxiety constitutes a serious public health concern. Cognitive behavioral therapy (CBT) is a gold standard treatment, preferred by families over pharmacological options, but barriers limit CBT accessibility. Modern CBT formats include varying levels of therapist involvement and differential technologies to overcome barriers, but little is known about their effectiveness in typical care settings, as well as in pediatric care. The Kids Formats of Anxiety Care Effectiveness study For Extending the Acceptability and Reach of Services (Kids FACE FEARS) trial addresses these gaps.

**Method:**

The Kids FACE FEARS trial was a multisite, pragmatic randomized trial comparing therapist-led CBT (telehealth, office-based, or hybrid) with guided internet-based CBT (self-administered/self-paced, with minimal therapist support) for treating anxiety in youth (7-18 years old) identified in pediatric care. English- and Spanish-speaking families were enrolled from high-volume, urban pediatric health care sites affiliated with major medical centers in 4 metropolitan regions. This article describes the study’s rationale, treatment conditions, participant recruitment, assessment schedule/strategy, and provider training/consultation.

**Discussion:**

Recent innovations have expanded CBT delivery options for pediatric anxiety. This is the first multisite randomized trial directly comparing CBT formats that draw on differential levels of therapist involvement and modes of technology. Sampling and study design features poise the Kids FACE FEARS trial to be one of the largest and most diverse/representative controlled trials of CBT for pediatric anxiety. In the context of evolving CBT delivery options, trial findings can inform patient-centered decision making and help tailor treatment selections for underserved youth with anxiety.

**Clinical trial registration information:**

Kids FACE FEARS Comparative Effectiveness Research; https://clinicaltrials.gov/study/NCT03707158

## Plain language summary

Cognitive behavioral therapy (CBT) is a “gold standard” treatment for youth anxiety, but barriers limit CBT accessibility. This study protocol describes novel ways of improving access in the Kids Formats of Anxiety Care Effectiveness study For Extending the Acceptability and Reach of Services (Kids FACE FEARS) trial—a multisite randomized trial comparing two CBT formats (ie, therapist-led CBT and guided internet-based CBT) with different levels of technology and therapist involvement. This study focused on English- and Spanish- speaking youth with anxiety in urban pediatric sites affiliated with major medical centers. Findings may better inform treatment recommendations in pediatric settings.

Pediatric anxiety and its sequelae constitute a very serious public health concern. Anxiety disorders are the most common psychiatric problems affecting children and adolescents,^1^, ^2^, ^3^, ^4^ with one-fifth of the population meeting criteria for an anxiety disorder by the time they reach adulthood.^3^ Pediatric anxiety is associated with considerable burdens, including school absenteeism, academic underachievement, social difficulties, family dysfunction, somatic symptoms, and frequent medical visits.^5^, ^6^, ^7^, ^8^ When left untreated, pediatric anxiety often persists into adulthood and can worsen with time, increaing risks for substance use, psychiatric impairments, occupational interference, and suicidal thoughts and behaviors.^9^, ^10^, ^11^, ^12^, ^13^, ^14^, ^15^

Pediatric anxiety rates have been rising across the past decade^16^, ^17^, ^18^, ^19^, ^20^ against a backdrop of increased political division and social unrest, widening disparities, harmful social media impacts, rising climate concern, and a global pandemic with far-reaching ripples and hardships. In the United States, minoritized status confers particular risk for anxiety, especially for youth of color, language-minority youth, and children of foreign-born caregivers.^21^, ^22^, ^23^, ^24^, ^25^ At the same time, minoritized youth are often underrepresented in anxiety research.

### Cognitive Behavioral Therapy Well Supported but With Limited Reach

Efficacious treatments for youth anxiety can decrease lifelong symptoms, functional burdens, costs, and the subsequent onset of comorbid problems (eg, depression, substance use).^26^, ^27^, ^28^, ^29^, ^30^ Cognitive behavioral therapy (CBT) is a gold standard psychological treatment for mild-to-moderate anxiety^31^, ^32^, ^33^, ^34^ and is preferred by families over pharmacological approaches.^35^

Dozens of large randomized trials indicate the majority of youth who experience anxiety achieve marked improvement after CBT, significantly outperforming waitlist, bibliotherapy, active support, and placebo comparators.^31^, ^32^, ^33^^,^^36^ In the landmark, multisite Child/Adolescent Anxiety Multimodal Study (CAMS),^34^ for example, 60% of youth treated with CBT were classified as treatment responders by independent evaluators (IEs) compared with 55% of youth treated with sertraline and only 23% of youth treated with placebo. For youth with severe anxiety, a combination of CBT plus sertraline (associated with an 80% responder rate) was required for response,^37^ although medication approaches introduce side effect concerns for families to consider. Recent work has also called into question whether added benefits observed from multimodal treatment reflect true augmentation effects or simply a placebo effect added to existing CBT gains.^38^

Despite the efficacy of CBT for youth anxiety, up to 80% of anxious youth do not seek or receive help.^3^ Among individuals with anxiety disorders who do receive care, the median delay from disorder onset to time of initial treatment contact ranges from 9 to 23 years.^39^ Such failures and delays in treatment utilization underscore major problems in the availability, accessibility, and acceptability of care. For many, traditional office-based care presents transportation obstacles, time demands that compete with work needs, childcare coordination challenges, and prohibitive costs and copayments.^40^ The relative unavailability of services in non-English languages causes linguistic disparities,^41^ and institutional mistrust and stigma associated with visiting a mental health clinic place office-based care out of reach for many.^42^^,^^43^ Additionally, for several years the COVID-19 pandemic fully shut down the majority of office-based services.

Service barriers disproportionately affect youth from minoritized communities, and anxious youth from such communities are particularly underserved. For example, anxious youth of color and in families with resource insecurity are less likely to receive anxiety services than non-Hispanic White youth^44^^,^^45^ and youth from resource-secure households.^45^^,^^46^ Also, anxious youth in immigrant households and in non–English-speaking households use mental health services less than anxious children of US-born caregivers and English-speaking households.^47^

### Promise of Technology-Based Strategies for Improving Access to Care

Technology-based strategies for extending the delivery of CBT to youth who experience anxiety have been supported^48^, ^49^, ^50^, ^51^, ^52^, ^53^, ^54^, ^55^, ^56^, ^57^, ^58^, ^59^ and show promise for overcoming treatment barriers. Research shows rapidly rising rates of household internet availability in the United States: 97% of people younger than 65 years of age now report regular internet use, and 85% report having household internet access.^60^ Although disparities in internet access persist, 91% of Black people and 86% of people earning <$30,000/year nonetheless report regular internet usage.^60^ Work remains for further expanding internet access, but these recent trends speak to the potential that internet-delivered CBT formats have for broadening the reach of care. Two leading internet-delivered approaches for extending CBT availability are telehealth and hybrid care and guided internet-based care.

### Telehealth and Hybrid Care

Telehealth formats and hybrid options (ie, mix of telehealth and office-based care) leverage synchronous telecommunications (typically videoconferencing) for the remote provision of live and interactive therapist-led care. Telehealth has been increasingly studied^48^, ^49^, ^50^, ^51^ as a means to overcome logistical challenges to traditional office-based CBT for youth anxiety and stigma about attending a mental health facility. Research in controlled settings and specialty clinics finds that telehealth options for a range of child mental health challenges can produce gains comparable to traditional office-based CBT.^48^, ^49^, ^50^, ^51^^,^^61^, ^62^, ^63^ In some trials, telehealth has outperformed traditional office-based care on key outcomes,^62^ likely due to the improved ecological validity afforded in telehealth by treating families in their natural spaces. Furthermore, relative to traditional office-based care, telehealth is associated with significantly reduced caregiver-perceived barriers to care^62^ and significantly improved session attendance, particularly for racial and ethnic minoritized youth.^64^ After years of research, telehealth entered the clinical mainstream during the COVID-19 pandemic,^65^, ^66^, ^67^ during which time it temporarily became the dominant mode of outpatient mental health care. In postpandemic times, telehealth still plays a prominent (albeit understudied) role in youth mental health care.

### Guided Online Care

Guided internet-based care (ie, self-administered/self-paced, with minimal therapist support) offers a computerized CBT delivery format that reduces therapist demands relative to both office-based care and telehealth, while affording greater family flexibility, agency, and control. As such, guided internet-based care not only addresses many of the same logistical care barriers as telehealth, but also can address person-power issues in the mental health workforce, inconsistencies in care quality across practice settings, cost issues, and, for some families, issues of mistrust about working directly with a health care professional. Accordingly, self-administered online CBT may in some cases be the preferred treatment mode. CBT lends itself well to digitization and standardization to reduce treatment drift due to its highly structured nature, and digital delivery may be particularly well suited to youth and younger families who avidly engage with media and technology. That said, attrition can be high in self-administered online care. Increasing studies find that some level of minimal/low-intensity human support is often needed to accompany self-administered online CBT—ie, guided internet-based CBT (iCBT)—to sustain patient motivation, promote adherence, and prevent disengagement.^68^^,^^69^ Several very strong self-paced, computerized CBT programs have been developed for the treatment of youth anxiety, and controlled trials have shown that many of these programs can produce sizable treatment gains, particularly when administered with some level of guided support.^52^, ^53^, ^54^, ^55^, ^56^, ^57^, ^58^, ^59^

### Gaps in Understanding How Various Pediatric Anxiety Treatment Formats Perform and Compare in Typical Care Settings

Despite great promise in the use of technology-based strategies relying on varying levels of therapist involvement to expand the reach of CBT for pediatric anxiety, much remains to be learned about how such modernized CBT formats perform in typical care settings. Furthermore, integrating or co-locating behavioral health services in pediatric settings can be an effective strategy for meaningfully extending the reach of evidence-based youth mental health care, but comparative effectiveness trials of technology-based anxiety treatments have not been conducted in pediatric care. In addition, little is known about what factors may facilitate vs challenge successful engagement with these modernized CBT formats in usual care settings and whether specific subpopulations of anxious youth may differentially benefit from these options. With regard to therapist-led telehealth and hybrid strategies for pediatric anxiety, most support has come from small-scale trials conducted in tightly controlled contexts and anxiety specialty clinics with highly selected samples and research therapists. Such work cannot speak to telehealth effectiveness or hybrid treatment under typical care circumstances and is underpowered to examine predictors of differential telehealth response. With regard to iCBT for pediatric anxiety, research to date has been conducted with predominantly non-Hispanic White and English-speaking samples, and most of the trials have been implemented in anxiety specialty clinics and/or research settings (with some exceptions^70^^,^^71^). Evaluating the effectiveness of iCBT in diverse populations under usual care conditions is critical for understanding the extent to which this format can truly expand the accessibility and acceptability of care and reach underserved populations. Further, clinical trials of iCBT have not included a therapist-led treatment comparison, rendering it hard to make informed comparisons across treatment formats and precluding an understanding of which CBT formats for pediatric anxiety work best for whom.

### Kids FACE FEARS Trial: Overview and Specific Aims

Building on the strong evidence supporting CBT for the treatment of mild-to-moderate pediatric anxiety, the present multisite trial was designed to examine the comparative effectiveness of modernized CBT delivery formats that have shown initial promise for expanding the reach of care. Specifically, the Kids FACE FEARS trial (Kids Formats of Anxiety Care Effectiveness study For Extending the Acceptability and Reach of Services) was a type 1 hybrid effectiveness and implementation pragmatic study designed to compare therapist-led CBT (telehealth, office-based, or hybrid) vs iCBT (with minimal therapist support) in usual care settings for the treatment of pediatric anxiety. Youth were universally screened for anxiety as part of routine primary or secondary pediatric care across health networks in 4 metropolitan regions. Youth with elevated anxiety were referred to integrated or co-located behavioral health teams in their hospital system and, if eligible and interested, were randomized to 1 of the 2 treatment comparators (which were provided in English and Spanish) and followed through a follow-up period. To maximize generalizability, exclusion criteria were minimal compared with previous large-scale randomized controlled trials of youth anxiety treatment.^34^ To observe treatment comparators under natural circumstances in general pediatric health care settings, study treatment was incorporated into the natural flow of care at each participating site (eg, clinical care was not funded by the study grant, research therapists were not used, anxiety specialty clinics were not involved in clinical care). Table 1 presents the specific aims and objectives of the Kids FACE FEARS trial.

**Table 1:** Specific Aims of the Trial

Aim	Domain	Objective
I	Comparative effectiveness	To compare acute and longer-term effectiveness of therapist-led CBT (telehealth, office-based, or hybrid) vs iCBT (with minimal clinician involvement) for treating youth anxiety identified in pediatric health care
II	Heterogeneity of treatment effects	To determine whether key factors predict or moderate differential treatment engagement or response, in turn informing treatment personalization for various patient subgroups
III	Implementation facilitators and barriers	To explore quantitative and qualitative data to understand perspectives, preferences, background factors, clinical and treatment engagement variables, and organizational factors that impede or facilitate implementation of, and patient engagement with, the comparators in pediatric health settings

Note: CBT = cognitive behavioral therapy; iCBT = guided internet-based CBT.

## Method

Institutional review boards at each participating site approved and oversaw the human subjects research aspects of the study, with the Boston Medical Center Institutional Review Board serving as the primary ethics review board for the trial. The trial was preregistered (ClinicalTrials.gov ID: NCT03707158). Throughout all stages of the Kids FACE FEARS trial, investigators engaged in collaborations with patient, parent, and community stakeholders who have experience (themselves or loved ones) with mental health challenges such as anxiety (ie, patient and family advisory council [PFAC]), as well as with treatment providers, clinical supervisors, and program administrators who are responsible for providing care to families. Investigators also engaged with a study advisory committee comprising members dedicated to improving access to evidence-based treatment and a data safety and monitoring board to further ensure patient safety and protection. These mutual partnerships with our stakeholder groups meaningfully informed each aspect of the study design, implementation, and dissemination.

### Study Setting

This multisite comparative effectiveness trial was conducted at high-volume, urban pediatric health care sites affiliated with 4 major medical centers (Johns Hopkins Hospital in Baltimore, Maryland; Boston Medical Center in Boston, Massachusetts; Nicklaus Children’s Hospital in Miami, Florida; and Seattle Children’s Hospital in Seattle, Washington). These sites were selected as clinical performance partners due to their roles in serving large numbers of diverse pediatric patients and providing a high volume of services in English and in Spanish, their collective representativeness of under-resourced pediatric health settings that serve diverse populations from a range of socioeconomic backgrounds, and the advantages offered in studying the treatment comparators in the context of academic hospital–community health center partnerships. For example, Boston Medical Center is the largest safety net hospital in New England with a large affiliated network of community health centers. Nicklaus Children’s Hospital is home to the largest pediatric teaching program in the Southeastern United States, and the majority of its patient population is Hispanic/Latine, reflecting the demographics of the South Florida community.

### Participation Flow

#### Overview of Study Timeline

**Figure 1:**
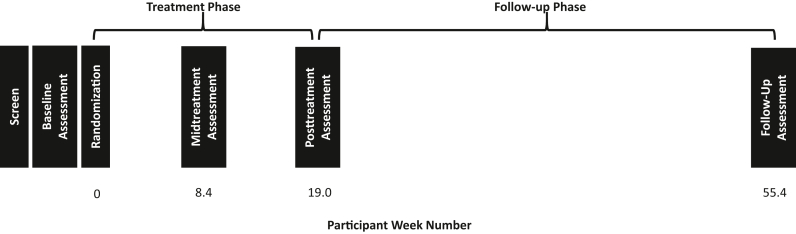
Overview of Study Timeline for Participants ***Note:****Week numbers reflect the mean number of actual weeks since baseline that participants in the Kids Formats of Anxiety Care Effectiveness study For Extending the Acceptability and Reach of Services (Kids FACE FEARS) trial completed assessments at each time point.*

Figure 1 presents the study timeline for participating families. Following initial screening and consent, families completed baseline assessment (week 0), and eligible participants were then randomized to 1 of the 2 treatment comparators. Participants were granted up to 20 weeks to complete their allocated treatment. The decision for a 20-week treatment window was informed by PFAC and stakeholder input urging that an appropriate and realistic time frame for care would be one that allows for missed sessions, holiday breaks, health-related cancellations, and attendance-interfering life events. Families were asked to complete mid-treatment assessment when they completed half of their allocated treatment or at week 8 (whichever came first). Families were invited to complete posttreatment assessment when they fully completed their allocated treatment program or at week 20 (whichever came first). All families were invited to complete a follow-up assessment at week 52.

#### Recruitment

Eligible and interested families across the participating pediatric health care networks were enrolled in the Kids FACE FEARS trial from November 11, 2019, to February 14, 2022. All participating primary and secondary pediatric care clinics used the 8-item Patient-Reported Outcomes Measurement Information System (PROMIS) Pediatric Short Form v2.0–Anxiety 8a,^72^, ^73^, ^74^, ^75^ the Pediatric Symptom Checklist–17 (PSC-17),^76^ Generalized Anxiety Disorder–7 (GAD-7),^77^ and/or the Patient Health Questionnaire–9 (PHQ-9)^78^ to initially screen for elevated anxiety. These screenings were part of standard practice at the participating sites. For clinics using only the PHQ-9 (which provides a depression score) for initial screening of potential internalizing problems, elevations were followed up with administration of a validated anxiety assessment. Youth in primary and secondary pediatric care settings who showed elevated anxiety scores were referred to integrated or co-located behavioral health teams in their hospital for information about the Kids FACE FEARS trial. Families at the participating primary and secondary pediatric care clinics who voiced concerns about youth anxiety (regardless of screener scores) were also referred to integrated or co-located behavioral health teams in their hospital for information about the trial. Health Insurance Portability and Accountability Act waivers were secured to access (but not store) screening data for the purposes of identifying and contacting potential study participants. The referral and permission were documented through the internal mechanism(s) of each pediatric health setting for referring families to ancillary services and case management. The overall goal was to align referral and consent as closely as possible with existing workflows to reduce staff/family burden and to support sustainability. Primary and secondary pediatric care providers and staff were educated about the study to increase project awareness and facilitate referrals. Sites were not required to receive consent to screen because screening was part of standard care. When a family was referred to the study, a Kids FACE FEARS staff member contacted them to inform them about the study, answer any questions, review eligibility, and enroll them if interested and eligible.

#### Randomization

Randomization assignments were made centrally at the study data coordination site. Families were assigned 1:1 to 1 of the 2 treatment conditions by randomization software programmed to stratify assignments by site (Baltimore, Boston, Miami, or Seattle), language of care (English or Spanish), and age (7-12 years or 13-18 years). Once a participating family’s baseline assessments were completed, the randomization software was programmed to send an automated push notification revealing their assigned condition simultaneously to the Kids FACE FEARS data management team (for data recording purposes) and to the research coordinators and clinical team at the participant’s site (to orient the family to their treatment condition).

#### Data Collection and Retention

Questionnaire data were collected online via REDCap (https://project-redcap.org/), an encrypted electronic data capture platform or via paper-and-pencil method when preferred by families and then entered into the REDCap platform and double-checked by staff. Given the aim to evaluate treatment engagement and performance under typical circumstances, families were not compensated for participating in treatment. To ensure generalizability and observe the comparators under natural conditions, treatment was not funded by the study or offered free of charge. Rather, treatment was covered via natural payment channels (eg, insurance and copayments). In contrast, families received staggered compensation for participation in study assessments that were not part of the treatment comparators under study. Automatic reminders, weekly emails, and phone calls from Kids FACE FEARS staff were also used to increase participation in study assessments. Families who did not complete baseline assessment were not randomized. Families who did not complete their assigned treatment were still invited to complete posttreatment and follow-up evaluations.

### Participants

#### Sample Size Determination

Power analysis to determine the appropriate sample size for the trial had to take into account data clustering and repeated measurements. Study data were clustered in multiple ways, resulting in nonindependent observations and inflated type I error. First, there were multiple observations per child due to the repeated measures design with 4 major assessment points. Second, there was additional clustering due to multiple children from each site. The intraclass correlation (ICC) is a quantitative estimate of clustering and allows for adjustment for nonindependence. Study investigators estimated an ICC of 0.5, meaning it was anticipated that 50% of the variation in the observations would be due to differences between individuals and between sites (the remaining 50% of the variation was assumed to be due to individuals varying in their responses over time). The design effect (DE) reflects the extent to which standard errors are deflated if clustering is ignored. The DE is equal to 1 + (m − 1) × ICC, where *m* is the number of repeated measures. Accordingly, with 4 time points and an assumed ICC of 0.5, the DE was calculated to be 2.5. Any sample size estimate must be multiplied by 2.5 to obtain a sample size appropriate for the observed clustering while maintaining nominal α and power.

The required study sample size was calculated via conventional methods for repeated measures/mixed models analysis. Required sample size is a function of the α (type I error rate) and β (1 − power) values. Required size per group is calculated as follows: n = 2 × (Z_1 −__α_ + Z_1 −__β_)^2^. Before adjusting for the DE, these computations found that n = 60 was required in each group for α = .05 (assuming familywise error rates = 0.05 across the study to account for multiple comparisons) and power = 0.8 (corresponding to β = .2). After adjusting for the DE of 2.5, this power analysis indicated that n = 150 was required per group (60 × 2.5), for a total sample N = 300.

The needed sample size of N = 300 was based on the primary comparative effectiveness tests of this study (aim I). That said, a sensitivity analysis was also conducted to evaluate the magnitude of effects that could be detected in moderation analyses examining heterogeneity of treatment effects (aim II). This was conducted with G∗Power (https://www.psychologie.hhu.de/arbeitsgruppen/allgemeine-psychologie-und-arbeitspsychologie/gpower) using the repeated measures analysis of variance (ANOVA) test. Repeated measures ANOVA is similar to a mixed model in some respects, but simpler and uniformly less powerful^79^; using the repeated measures ANOVA, therefore, offers a conservative estimate of effects able to be detected with each mixed model. With N = 300, α = .05, power = 0.8, 2 treatment conditions, 4 measurements, and a correlation between repeated measures of 0.5, the trial was powered to detect moderation effects as small as *d* = 0.068. This is 7% of a standard deviation difference and a very small effect.

#### Eligibility

To optimize generalizability, eligibility criteria were intentionally inclusive, and exclusion criteria were minimal (Table 2). English- and Spanish-speaking families were eligible, there were no comorbid diagnosis rule-outs, autistic children were eligible unless they had very severe challenges (eg, complete absence of verbal communication unrelated to anxiety), and youth receiving pharmacological care were included as long as they were taking stable doses. For patient safety, youth requiring higher levels of care were excluded and referred for appropriate services. The decision to include families fluent in either English or Spanish was made to improve the generalizability of trial findings, relative to existing research on the treatment of youth anxiety; recruit a sample that would be more representative of the general US population and the diverse range of anxious youth seen in clinical practice; and provide treatment-related findings that would also be informative to the estimated 33.3% of mental health treatment facilities that provide services in Spanish.^80^

**Table 2:** Eligibility Criteria

Inclusion criteria	Exclusion criteria
• Elevated child/adolescent anxiety o Operationalized as T score on PROMIS Pediatric Short Form v2.0–Anxiety 8a ≥55^a^ • Child age 7-18 y (inclusive) at time of screening • Child and caregiver(s) fluent in English or Spanish • If taking medication for emotional problems, child must be on a stable dose o Operationalized as no adjustments to prescription for ≥8 wk)	• Severity requiring higher level of care, as defined by any of the following: o STB with active plan or STB that required a higher level of care within past 6 mo (eg, inpatient, partial hospitalization) o Anxiety-related absences ≥50% of school days over the past month^b^ o Substance use that required emergency services or inpatient/partial hospitalization within past 3 mo o Clinician determined child requires higher level of care than outpatient services • History of diagnosed autism spectrum disorder or intellectual disability with severe challenges and needs for support (eg, complete absence of verbal communication unrelated to anxiety) • Currently engaged in CBT or plan to continue other therapy for anxiety during study treatment phase • Child is a ward of the state

Note: CBT = cognitive behavioral therapy; PROMIS = Patient-Reported Outcomes Measurement Information System; STB = suicidal thoughts and/or behavior.

a Eligible if either the Pediatric Self-Report or the Parent Proxy Report score was ≥55.

b If summer, attendance during last month of previous school year was considered.

Several factors informed the decision to not consider formal *DSM* or *ICD* diagnoses as part of study inclusion or exclusion. Eligibility criteria were intentionally relaxed relative to previous large-scale trials (eg, ^34^) to better approximate the full range of anxious youth in need of care. Inclusion criteria for this trial required youth to have elevated anxiety levels, but did not require formal anxiety disorder diagnoses, given the high number of anxious individuals in need of care who miss diagnostic thresholds by *DSM* or *ICD* technicalities,^81^^,^^82^ especially in medical populations.^83^, ^84^, ^85^ Further, comorbid diagnoses did not preclude eligibility in the Kids FACE FEARS trial (as long as they did not acutely require a higher level of care such as inpatient or partial hospitalization), given research showing such requirements can exclude up to half of children seeking care from research trials.^86^ The decision to not consider *DSM* or *ICD* diagnoses as part of study eligibility is consistent with concerns about the poor reliability and differentiation of pediatric anxiety disorder diagnoses^87^, ^88^, ^89^, ^90^ and associated trends in clinical science priorities moving away from formal diagnoses.^91^^,^^92^ Moreover, there is evidence that clinicians rarely use structured diagnostic interviews outside of research contexts,^93^ rendering findings based on structured diagnostic interviews poorly aligned for informing clinical practice. Finally, not including structured research-based diagnostic interviews in the trial’s assessment protocol addressed the need to minimize participant burdens and assessment demands in a pragmatic effectiveness trial. Reducing the assessment time that was focused on diagnostic nuances also afforded opportunity to instead assess several important factors that have previously been neglected in large-scale clinical trials for the treatment of child anxiety (eg, adverse childhood events, experiences of discrimination, mental health stigma).

### Interventions and Comparators

The Kids FACE FEARS trial tested 2 CBT delivery strategies for pediatric anxiety: therapist-led CBT and iCBT (with minimal therapist support).

#### Comparator 1: Therapist-Led CBT for Youth Anxiety

An extensive body of controlled studies supports therapist-led CBT for anxious youth and their families, with therapist-led CBT conventionally considered a gold standard psychological treatment for anxious youth.^31^, ^32^, ^33^, ^34^ Therapist-led CBT has traditionally entailed office-based care in which a therapist directly trains youth (and often caregivers) in anxiety management skills, then guides youth in planning and participating in graded exposure tasks in which they approach increasingly feared situations. In the landmark Child/Adolescent Anxiety Multimodal Study (CAMS) trial, approximately 60% of youth treated with therapist-led CBT were classified as treatment responders by masked IEs.^34^ In more recent years, telehealth formats of therapist-led CBT for youth anxiety have received considerable and comparable research support.^48^, ^49^, ^50^, ^51^

Although a number of established and highly overlapping therapist-led CBT programs for youth anxiety exist, the well-supported Cool Kids CBT suite for youth anxiety^94^^,^^95^ was selected for several important reasons. First, Cool Kids includes all the gold standard CBT components for treating youth anxiety and has an extensive research base.^96^, ^97^, ^98^ Second, Cool Kids is a time-limited and relatively brief suite of treatment programs delivered across approximately 3 months, which accommodated the PFAC and stakeholder advisor suggestion for relatively short-term treatment. Third, the Cool Kids suite comprehensively includes developmentally tailored versions for the full age range of youth included in the study (7-18 years). Fourth, the Cool Kids treatment suite has a documented history of successful implementation when rolled out on large-scale levels.^98^^,^^99^ Fifth, a parallel self-administered online version of Cool Kids teaches youth the same anxiety management skills as the therapist-led version and in a comparable number of sessions/modules, thus allowing for well-matched and time-equitable comparisons. Finally, all the Cool Kids programs are all available in English and in Spanish.

The standard Therapist-Led Cool Kids Therapy for Anxiety (ages 7-12)^94^ is a structured, evidence-based, 10-session program for treating youth anxiety in middle childhood and entails 50-minute weekly sessions over 10 weeks with children and families. Therapists teach youth and caregivers core CBT components including anxiety psychoeducation, cognitive/coping skills for thinking more realistically and adaptively, problem-solving skills, and graded exposures to anxiety-provoking situations. Caregivers and youth are taught skills for managing anxiety and for reducing avoidant behaviors. Caregivers are included in all sessions (adjusted developmentally) and are taught parenting skills plus personal anxiety management. The program is supported by a structured therapist manual and includes developmentally tailored child and parent workbooks. Cool Kids is available in many languages, including English and Spanish, and has been adapted slightly for different age groups. The Therapist-Led Chilled Therapy for Anxiety (ages 13-18 years) is an adolescent version of Cool Kids and includes a workbook designed specifically for adolescents; in this version, teens take a more central role in managing their anxiety, and caregivers do not participate in all of the sessions. This version also includes 10 sessions.

#### Comparator 2: iCBT for Youth Anxiety (With Minimal Therapist Support)

To expand the accessibility and reach of CBT for youth who experience anxiety, a suite of self-paced, online, multimedia, CBT-based programs for youth anxiety was developed to run parallel to the therapist-led Cool Kids program. This online suite of programs covers identical content in a largely self-administered format. Similar to therapist-led Cool Kids, the Cool Kids Online suite of programs comprises separate developmentally tailored online programs, 2 of which were used in the present trial, depending on the age of the child: Cool Kids Online for children ages 7 to 12 years and Chilled Out for youth ages 13 to 18.^55^^,^^100^^,^^101^ These programs consist of 8 modules that are to be completed approximately once a week allowing for well-matched and time-equitable comparisons with therapist-led CBT. The online programs have already been used in numerous countries and are available in multiple languages, including Spanish. The programs include the same core CBT components as the therapist-led Cool Kids programs; feature interactive, engaging formats; and include video and audio clip examples of how to implement skills. In Cool Kids Online, caregivers take on the role of their child’s coach, helping them put their skills into practice, and learn helpful ways of responding to their child’s anxiety. In the Chilled Out adolescent online version, the teen takes primary responsibility for program completion, and there are features designed to specifically increase teen engagement—eg, real teens are featured in the videos, teens demonstrate skills for managing anxiety, and a playlist menu allows teens to revisit content and cover skills at their own pace. The programs were originally developed in Australia. For both Cool Kids Online and Chilled Out, Americanized versions of the programs were used that include videos with diverse representation (eg, American children, families, and experts; broader representation of individuals of color; removed Australian accents or vernacular).

To foster supportive accountability and engagement,^68^^,^^69^ families in this condition received brief phone check-in calls (ie, <15 minutes) from a therapist every other week. To ensure these brief calls did not drift into treatment sessions, therapists worked from a conversation guideline to standardize calls (with space for flexibility as appropriate). Therapists were instructed to begin each check-in call with an explanation of the purpose of the call—ie, to see how their use of the online modules was going, to find out whether the youth or caregiver had any questions, to support the family with the program, and to make a plan for continued practice. Therapists then checked in briefly about each of the following components:

1. Engagement with online program and activity completion: “Were you able to log into the online program? If not, what got in the way?”
2. Identification of current difficulties/barriers, including technical or motivational challenges: “Did anything get in the way of completing activities or practice? Did you experience any difficulties as a parent with respect to your child’s anxiety management?”
3. Brainstorming possible solutions to identified barriers: “Let’s see if we can problem-solve together and come up with some solutions to help you complete materials this week.”
4. Review of anxiety and caregiver progress: “Has your/your child’s level of anxiety changed since beginning the program?
5. Technical questions about content: “Do you have any specific questions about the material that you learned in the modules you completed?”
6. Assignment/plan for next 2 weeks, providing encouragement and praise for even small successes.
7. Confirmation of the next call.

### Therapist Training and Ongoing Consultation

Existing therapists and clinical supervisors from across the participating pediatric health networks (ie, clinical staff not employed by the research trial and not working in anxiety specialty settings) were trained by the study team to deliver CBT for youth anxiety. Consistent with best practices for promoting quality implementation of evidence-based treatment,^102^, ^103^, ^104^, ^105^ a multicomponent strategy was used that involved training workshops for therapists and supervisors before their participation in the trial, followed by asynchronous online resources and ongoing small group consultation of therapists and supervisors. Training entailed 1 full-day (8 hour) training or 2 half-day trainings consisting of didactic components and active teaching approaches (eg, live role plays, demonstrations of core skills, small breakout groups, interactive video presentations, opportunities for therapists to actively practice using specific CBT skills to treat child anxiety). These trainings also included a demonstration of the iCBT (including illustrative video clips and log-in/access tutorials) and incorporated instruction on conducting check-in calls for iCBT. Therapists and supervisors were given an iCBT check-in call conversation guide/script (see “Comparator 2: iCBT for Youth Anxiety (With Minimal Therapist Support”) and were taught how to navigate common questions. After each training, therapists and supervisors completed posttraining knowledge quizzes. Any therapist who scored <80% on the posttraining knowledge quiz received individualized follow-up training and support before treating study patients.

After each training, therapists and supervisors were provided access to asynchronous online resources to support their delivery of the treatment comparators, including session outlines, training recordings, and booster supports as needed. A website with helpful resources was developed to further support therapists as they implemented treatment. This website included treatment demonstration videos, treatment module summaries, and therapist training slides. A guided refresher course containing audio and video training demonstration videos and interactive training activities was created and included on the website.

In addition to routine supervision that may have been normally provided in their hospital or clinical network, providers at the study performance sites also participated in regularly held group consultation videoconferencing calls led by pediatric anxiety treatment experts. All providers who attended a training workshop were assigned to a recurring consultation call group. Therapist consultation calls were held twice monthly and designed to support therapists, prevent therapist drift, and afford education and scaffolding to providers. Approximately 4 to 6 therapists were assigned to each call group, and the remote format enabled the call groups to contain a mix of therapists from across the study sites. Calls lasted 1 hour each and were used to clarify and role-play therapy skills, answer any treatment questions, and support therapists as they implemented the treatments. Site supervisors also participated in separate, monthly supervisor group consultation calls, also led by pediatric anxiety treatment experts.

Each consultation call was structured to formally review a specific rotating skill or topic, including psychoeducation, fear hierarchies/exposures, cognitive strategies, cultural considerations, parenting factors, check-in calls, homework compliance, coping skills, treatment flexibility,^106^ COVID-19-related anxiety, school anxiety, developmental considerations, and technology-related issues. Each call also included a “therapist spotlight,” in which 1 therapist had an opportunity to receive more individualized attention and in-depth consultation about a case. These therapist spotlights gave call leaders the opportunity to clarify and correct any potential problems with treatment delivery and to provide individualized follow-up training and therapist support as needed. Therapist spotlights also provided opportunities for peer input and for therapists to learn from the experience and perspectives of one another.

### Overview of Measures

All measures and interviews were administered in English or Spanish, depending on participant preference. Consistent with a recommended multi-informant assessment strategy,^107^^,^^108^ measurement of outcomes included caregiver-report questionnaires, youth-report questionnaires, therapist-report questionnaires, ratings of IEs masked to assigned treatment conditions, and administrative logs. To maximize generalizability and rigor while minimizing patient and clinic burdens, priority was placed on supported measures that were brief and free of charge in the public domain. Consistent with growing recognition that clinical interventions are intended to impact a range of domains of functioning (rather than a single narrow domain),^109^^,^^110^ a set of outcomes were included to consider multiple aspects of treatment response.

Although attempts were made to collect data from both caregivers and youth across assessment points for all families, to minimize participant burden and maximize engagement, only caregiver reports were required to randomize children ages 7 to 12 years, and only youth self-reports were required to randomize adolescents ages 13 to 18 years. Furthermore, 7-year-old participants were not administered self-report questionnaires due to concerns from the PFAC and scientific advisory panel (and corroborated by scientific literature^111^, ^112^, ^113^) about the reliability and validity of anxiety self-reports in children this age. These children were given self-report forms at later assessment points if they turned 8 during the trial. In addition, although youth self-reports of primary and secondary clinical outcomes were administered to all participants ≥8 years old, youth self-reports of a subset of treatment variables and other variables were administered only to youth ≥13 years old, given recommendations from the trial’s PFAC and scientific advisory panel to minimize the assessment burdens outside of clinical outcomes for youth ≤12 years old and concerns from these advisory groups about the ability of younger children to accurately self-report regarding some of these variables (eg, their own treatment histories, family treatment barriers). Measures included in the Kids FACE FEARS trial can be sorted into 4 groups: primary outcomes; secondary outcomes; treatment variables; and study covariates, predictors, and other included measures.

### Measures for Primary Outcomes

Primary outcomes were anxiety and impairment, treatment responder/remission status, and patient-centered outcomes (perceived effectiveness, satisfaction) (Table 3).

**Table 3:** Primary and Secondary Outcomes

	Domain	Measure	Informant(s)	Assessment point			
				Baseline	Mid-treatment	Posttreatment	1-Year follow-up
Primary outcomes	Youth anxiety symptoms	PROMIS Pediatric Short Form v2.0–Anxiety 8a	C, Y	X	X	X	X
	Anxiety-related impairment	CALIS	C, Y	X	X	X	X
	Treatment responder and remission status	PARS	IE	X		X	
	Primary patient-centered outcomes						
	Family-perceived effectiveness	Perceived effectiveness scale	C			X	X
	Treatment satisfaction	Satisfaction scale	CS, Y		X	X	
Secondary outcomes	Broader youth psychopathology						
	Internalizing problems	PSC-17	C, Y	X	X	X	X
	Externalizing problems	PSC-17	C, Y	X	X	X	X
	Attention problems	PSC-17	C, Y	X	X	X	X
	Youth sleep difficulties	Sleep item	C, Y	X	X	X	X
	Caregiver internalizing symptoms						
	Depression symptoms	DASS-21	CS	X	X	X	X
	Anxiety symptoms	DASS-21	CS	X	X	X	X
	Stress symptoms	DASS-21	CS	X	X	X	X
	Therapist perceptions of treatment response						
	Therapist-perceived effectiveness	Perceived effectiveness scale	T			X	
	Therapist-perceived improvement	CGI-I Scale	T			X^a^	

Note: C = caregiver report of child; CALIS = Child Anxiety Life Interference Scale; CGI-I = Clinical Global Impressions–Improvement; CS = caregiver self-report; DASS-21 = Depression, Anxiety, Stress Scale–21; IE = independent evaluator report (masked to treatment condition); PARS = Pediatric Anxiety Rating Scale; PSC-17 = Pediatric Symptom Checklist–17; PROMIS = Patient-Reported Outcomes Measurement Information System; T = therapist report; Y = youth self-report (administered to youth ≥8 years).

a Measure also administered after each treatment visit/check-in call.

#### Anxiety Symptoms

The PROMIS Pediatric Short Form v2.0–Anxiety 8a was used to assess severity of youth anxiety across time. Caregiver reports were collected via the parent-proxy form, and youth self-reports were collected via the pediatric form for youth ages ≥8 years. Across psychometric studies, the measure has exhibited strong reliability, structure, and validity in the measurement of youth anxiety severity.^72^, ^73^, ^74^, ^75^ The PROMIS Pediatric Short Form v2.0–Anxiety 8a is also free, brief (8 items), and publicly accessible, which minimizes patient and clinic burdens and positions the measure for use in under-resourced settings and other typical care settings.^72^ Raw total scores are converted to T scores normed for age and sex.

#### Anxiety-Related Impairment

The Child Anxiety Life Interference Scale (CALIS)^114^ is a measure of life interference and impairment associated with youth anxiety. Items are each rated on a 5-point Likert-style scale (0 = “not at all”; 4 = “a great deal”) and summed to generate a total interference score. Parents and youth ≥8 years old completed separate CALIS forms to offer distinct informant accounts of anxiety-related life impairment. The parent form consists of 16 items (scoring range: 0-64), and the youth form consists of 9 items (scoring range: 0-36). The CALIS parent and youth forms have exhibited strong psychometric properties in previous research.^114^^,^^115^

#### Treatment Responder and Remission Status

The Pediatric Anxiety Rating Scale (PARS)^116^ was used to independently evaluate clinical significance across the treatment conditions and to benchmark study findings against previously conducted randomized controlled trials on youth anxiety.^34^ The PARS is a well-supported, clinician-rated instrument for assessing the frequency and severity of anxiety symptoms associated with common anxiety disorders in children between the ages of 6 and 17 years.^116^^,^^117^ It consists of a 50-item symptom checklist followed by 7 global items each rated on a 6-point (0-5) scale. Six of the global items are summed to generate a PARS total score (range: 0-30). PARS total score reductions of 35% or more from baseline to posttreatment are interpreted as reflecting PARS treatment response; score reductions of 50% or more are interpreted as reflecting anxiety remission.^117^ For the present study, IEs masked to treatment condition conducted PARS interviews at baseline and at posttreatment with caregivers and youth together and then generated scores based on the pooled information.

#### Primary Patient-Centered Outcomes

To assess caregiver-perceived effectiveness, caregivers were asked on a 7-point scale at mid-treatment and again at posttreatment: “How effective do you think the program [has been/was] in treating your child’s anxiety?” (0 = “very ineffective”; 3 = “somewhat effective”; 6 = “very effective”). In addition, caregivers and youth ≥8 years old completed a patient-adapted version of the Clinical Global Impressions–Improvement (CGI-I) Scale^118^ to characterize their judgment of treatment-related improvement, relative to the child’s baseline presentation. Consistent with the standard CGI-I, scores of 1 (“very much improved”) or 2 (“much improved”) were interpreted as reflecting “caregiver-perceived treatment response” (for caregiver report) or “youth-perceived treatment response” (for youth self-report).

To assess treatment satisfaction, caregivers and youth ≥8 years old completed a satisfaction scale that had them rate 3 items on scales of 0 to 3 at mid-treatment and posttreatment: “Overall, how satisfied [have you been/were you] with the services that [your family/you] received?” (0 = “quite dissatisfied”; 3 = “very satisfied”); “Would you recommend this program to a friend if they [had a child with/had] anxiety?” (0 = “no, definitely not”; 3 = “yes, definitely”); and “How pleased [have you been/were you] with how this program has helped [your child/you] with anxiety?” (0 = “quite displeased”; 3 = “very pleased”). These items were averaged for each informant to respectively generate caregiver and youth total satisfaction scores. For further interpretation, mean scores ≥2 were interpreted as satisfied, and mean scores <2 were interpreted as dissatisfied.

### Measures for Secondary Outcomes

Secondary outcomes focused on broader youth psychopathology, youth sleep difficulties, caregiver internalizing symptoms (ie, depression symptoms, anxiety symptoms, and stress symptoms), and therapist perceptions of treatment response (Table 3).

#### Broader Youth Psychopathology

The PSC-17^79^ is a brief questionnaire developed to identify child and adolescent emotional and behavioral challenges in pediatric and primary care settings. The measure has shown strong validity and reliability across diverse samples of youth.^119^, ^120^, ^121^ PSC-17 subscales separately assess internalizing problems, externalizing problems, and attention problems. For the present study, caregivers and youth ≥8 years old completed separate PSC reports, and all 3 subscales were included.

#### Youth Sleep Difficulties

Caregivers were asked to rate on a 5-point scale the frequency with which the following statement applied to their child: “In the past 7 days, my child has been having sleep-related difficulties (for example, difficulty falling asleep or sleeping through the night)” (0 = “never”; 2 = “sometimes”; 4 = “always”). Youth ≥8 years old were similarly asked to rate on a 5-point scale the frequency with which the following statement applied to them: “In the past 7 days, I have been having sleep-related difficulties (for example, difficulty falling asleep or sleeping through the night)” (0 = “never”; 2 = “sometimes”; 4 = “always”).

#### Caregiver Internalizing Symptoms

The Depression, Anxiety, and Stress Scales–21 (DASS-21)^122^^,^^123^ is an adult self-report of negative emotional states that has shown strong psychometric properties.^123^^,^^124^ Respondents rate their experiences across 21 items on a 4-point severity/frequency scale ranging from 0 (“never”) to 3 (“always”). DASS-21 subscales assess depression, anxiety, and stress separately. All 3 subscales were used in the present study.

#### Therapist Perceptions of Treatment Response

To assess “therapist-perceived effectiveness,” therapists were asked the rate following statement at posttreatment on a 7-point scale: “How effective do you think the program was for treating this child?” (0 = “very ineffective”; 3 = “somewhat effective”; 6 = “very effective”). In addition, after each treatment session (for therapist-led CBT cases) or check-in call session (for iCBT cases), therapists completed the CGI-I^118^^,^^125^ to characterize their session-by-session judgment of the extent of treatment-related improvement, relative to the child’s baseline presentation. Therapists again completed a CGI-I at posttreatment.

### Measures for Treatment Variables

Treatment variables assessed in the Kids FACE FEARS trial focused on treatment preferences and expectancies, the scope and content of each session and check-in call, treatment fit and flexibility, treatment engagement and barriers, and therapeutic alliance (Table 4).

**Table 4:** Treatment Variables

Domain	Measure	Informant(s)	Assessment point				
			Baseline	After first session	After each session	Mid-treatment	Posttreatment
Treatment preferences and expectancies							
Treatment preference	Treatment preferences and expectancies survey	CS, Y	X				
Anticipated treatment comfort	Treatment preferences and expectancies survey	CS, Y	X				
Anticipated treatment comprehension difficulties	Treatment preferences and expectancies survey	CS, Y	X				
Anticipated treatment scheduling difficulties	Treatment preferences and expectancies survey	CS, Y	X				
Therapist-anticipated treatment effectiveness	Early treatment expectations form	T		X			
Therapist-anticipated participatory engagement	Early treatment expectations form	T		X			
Therapist-anticipated therapeutic alliance	Early treatment expectations form	T		X			
Therapist case-specific self-efficacy	Early treatment expectations form	T		X			
Scope and content of treatment sessions/check-in calls	Session summary form	T			X		
Treatment fit and flexibility	Session summary form	T			X		
Treatment engagement and barriers							
Attendance	Administrative data	A			X		
Homework engagement	Session summary form	T			X		
Treatment completion	Administrative data	T					X
Child participatory engagement	Session summary form	T			X		
Caregiver participatory engagement	Session summary form	T			X		
Comprehension difficulties	Treatment barriers survey	CS, Y				X	X
Difficulties making time for treatment	Treatment barriers survey	CS, Y				X	X
Treatment discomfort	Treatment barriers survey	CS, Y				X	X
Technology treatment challenges (family report)	Technological experiences and reactions scale	CS, Y				X	X
Technology treatment challenges (therapist report)	Technological experiences and reactions scale	T			X		
Therapeutic alliance (family report)	Perceptions of therapeutic alliance scale	C/CS, Y				X	X
Therapeutic alliance (therapist report)	Perceptions of therapeutic alliance scale	T					X

Note: A = administrative data; C = caregiver report of child; CS = caregiver self-report; T = therapist report; Y = youth self-report (administered to youth ≥13 years).

#### Treatment Preferences and Expectancies

Before randomization, caregivers and youth ≥13 years old completed a treatment preferences and expectancies survey designed for this study. Respondents indicated whether they would prefer to receive therapist-led CBT (coded 0) or iCBT (coded 2) or whether they had no preference between the 2 treatments (coded 1) (treatment preference). Respondents also rated each treatment on anticipated treatment comfort, anticipated treatment comprehension difficulties, and anticipated treatment scheduling difficulties on scales of 0 to 6.

Data were also collected on therapist’s early treatment expectations for each family. Specifically, after the first clinical encounter with an assigned family (ie, first session for therapist-led CBT families; first check-in call for iCBT families), therapists completed an early treatment expectations form developed for the present study. This form had therapists indicate their level of agreement with the following item on a scale from 0 (“very ineffective”) to 6 (“very effective”): “Thinking about the treatment course ahead, how effective do you think the child’s assigned treatment condition will be in treating this child’s anxiety?” (anticipated treatment effectiveness). To measure therapist’s anticipated effectiveness of the nonassigned treatment, therapists also used the same scale to indicate their level of agreement with the following statement: “If instead of [therapist-led CBT/iCBT], this family had been assigned to iCBT/therapist-led CBT], how effective do you think that treatment would be in treating this child’s anxiety?” (anticipated effectiveness of nonassigned treatment).

The early treatment expectations form also assessed therapists’ early expectations about treatment engagement for each family. Specifically, after their first clinical encounter with an assigned family*,* therapists were asked to respond to the following items on a scale from 0 (“to a small extent”) to 4 (“to a very great extent”): “Thinking about the treatment course ahead, to what extent do you expect this child will actively participate in their assigned course of treatment?” (anticipated child participatory engagement); “Thinking about the treatment course ahead, to what extent do you expect this child’s caregiver(s) will actively participate in their assigned course of treatment?” (anticipated caregiver participatory engagement).

The early treatment expectations form also assessed therapists’ early expectations about treatment alliance for each family. Specifically, after their first clinical encounter with an assigned family, therapists were asked to respond to the following items on a scale from 0 (“never”) to 6 (“always”): “Across treatment, I think this child and I will work well together” (anticipated therapist–child collaboration) and “Across treatment, I think this child’s caregiver(s) and I will work well together” (anticipated therapist–caregiver collaboration). Therapists used the same scale to rate the extent to which they predicted they would enjoy working with the child (anticipated therapist–child bond), the extent to which they predicted the child would enjoy working with them (anticipated child–therapist bond), the extent to which they predicted they would enjoy working with the child’s caregiver(s) (anticipated therapist–caregiver bond), and the extent to which they predicted the child’s caregiver(s) would enjoy working with them (anticipated caregiver–therapist bond).

Finally, the early treatment expectations form was used to assess therapist case-specific self-efficacy with regard to implementing the assigned treatment. Specifically, after their first encounter with a family, depending on the family’s treatment assignment, the therapist was asked to rate how well they predicted they would be able to conduct [therapist-led CBT/iCBT] on a scale of 0 (“very limited”) to 4 (“very high”).

#### Scope and Content of Treatment Sessions and Check-In Calls

After each treatment session (for therapist-led CBT cases) or check-in call session (for iCBT cases), therapists completed a session summary form that was developed for the present study. On this form, therapists indicated who participated in the session (eg, youth, caregiver, other), the language in which the session was held (English, Spanish, or both), whether the session began on time (and if not, how late it began), the length of the session, and the format of the session (eg, in-office, phone, videoconference). Therapists in each condition also checked off all of the content and topics that were covered in the session they just held, using the following list of options: psychoeducation; detective/realistic thinking; fear hierarchies; future exposure practice; in-session exposure practice; rewards and reinforcement; parenting issues related to youth anxiety; additional coping skills (eg, problem-solving, social skills, assertiveness, relaxation techniques); non-CBT strategies (eg, interpreting the meaning of symptoms, interpreting the meaning of child’s play or artwork); discussion of issues not directly related to anxiety (eg, major family transitions, conflicts other than co-occurring non-anxiety problems); review of previous homework; assignment of new homework; and addressing barriers to treatment/homework. For therapist-led CBT cases, therapists also indicated the session number(s) in the protocol that were covered in that session.

#### Treatment Fit and Flexibility

As part of the session summary form completed by the therapist after each treatment session (for therapist-led CBT cases) or check-in call session (for iCBT cases), therapists indicated their level of agreement with the following statement on a scale from 0 (“not at all”) to 6 (“extensively”): “To what extent was the content covered this [session/check-in call] a fit to this child’s clinical presentation and individual needs?” To assess needs for treatment flexibility, therapists also indicated after each session their level of agreement with the following statement on a scale from 0 (“not at all”) to 6 (“extensively”): “How much did you need to tailor or adapt the content or structure of this session because of the child’s clinical presentation and individual needs?” Moreover, as part of the session summary form, therapists selected the reason(s) they may have covered content outside of the treatment protocol in the session they just held from a list of options (if relevant):

1. Had to address a clinical emergency that made them concerned about immediate safety
2. Needed to address a topic of the week that did not directly relate to the focus of the treatment protocol (eg, family conflict, school trouble)
3. Had to address a co-occurring/co-presenting issue other than child anxiety (eg, attention-deficit/hyperactivity disorder, medical problem)
4. Needed to engage in an alliance-building activity
5. Needed to address treatment resistance
6. Other (describe)

#### Treatment Engagement and Barriers

Therapists completed weekly logs reporting whether study families on their caseloads attended their scheduled sessions or support calls and whether they completed the homework assigned in their previous session or support call. These data were used to characterize treatment attendance and homework engagement. Treatment completion was defined for therapist-led CBT as attending 10 treatment sessions and for iCBT as attending 4 support calls. For iCBT families, administrative backend data were also collected from the central server to further assess user/usage analytics.

To assess session-by-session participatory engagement, as part of the session summary form completed by the therapist after each treatment session (for therapist-led CBT cases) or check-in call session (for iCBT cases), therapists indicated their response to the following question on a scale from 0 (“not at all”) to 6 (“extensively”): “To what extent did you feel that this child was engaged in today’s [session/check-in call]. For example, did the child appear motivated and committed to improving, and was the child actively participating in the [session/call]?” (child participatory engagement). When appropriate, a parallel item was asked of therapists regarding the caregiver’s engagement in that session (caregiver participatory engagement). At posttreatment, therapists responded to modified versions of these items to report on participatory engagement across the entire course of treatment.

To assess comprehension difficulties, caregivers and youth ≥13 years old across both conditions were asked at mid-treatment and again at posttreatment: “How hard has the intervention been for [your family/you] to understand?” (0 = “never hard”; 3 = “sometimes hard”; 6 = “very hard”). To assess difficulties making time for treatment, caregivers and youth ≥13 years old were asked at mid-treatment and again at posttreatment: “How hard has it been for [your family/you] to [make your schedule work for treatment sessions/find time to work on and complete the computer-based treatment modules online]?” (0 = “never a problem”; 3 = “sometimes a problem”; 6 = “often a problem”). To assess treatment discomfort, caregivers and youth ≥13 years old were asked at mid-treatment and again at posttreatment: “How comfortable [has your family/have you] felt when [attending treatment sessions/completing the computer-based treatment modules online]?” (0 = “very comfortable”; 3 = “sometimes comfortable”; 6 = “very uncomfortable”).

The Technological Experiences And Reactions Scale (TEARS)^126^ was used to specifically assess technology-based treatment challenges. TEARS is a brief questionnaire that measures disruptions and patient frustrations with telehealth sessions and digital mental health. Psychometric research has demonstrated the reliability and validity of the measure. At mid-treatment and again at posttreatment, therapist-led CBT participants (most of which completed sessions via telehealth) and iCBT participants used TEARS to rate the extent to which technology issues and time spent addressing technology-related issues took away from the quality of the intervention, frustrated them, and/or interfered with treatment understanding. For families in both conditions, therapists also completed the therapist-report TEARS at the conclusion of each session/check-in call and again at posttreatment. In addition to TEARS, therapists and families reported basic information on how families were logging in and engaging with the treatment, and the devices they were using.

#### Therapeutic Alliance

Caregivers were asked at mid-treatment and again at posttreatment to respond to the following items on a caregiver perceptions of therapeutic alliance scale from 0 (“never”) to 6 (“always”): “I think the therapist and I [work/worked] well together to help with my child’s anxiety” (caregiver–therapist collaboration); “I feel like I [like/liked] the therapist” (caregiver–therapist bond); “I feel like the therapist [likes/liked] me” (therapist–caregiver bond); “I feel like my child [likes/liked] the therapist” (child–therapist bond); “I feel like the therapist [likes/liked] my child” (therapist–child bond).

Youth ≥13 years old were asked at mid-treatment and again at posttreatment to respond to the following items on a youth perceptions of therapeutic alliance scale from 0 (“never”) to 6 (“always”): “I think the therapist and I [work/worked] well together to help with my anxiety” (child–therapist collaboration); “I feel like I [like/liked] the therapist” (child–therapist bond); and “I feel like the therapist [likes/liked] me” (therapist–child bond).

Therapists were asked at posttreatment to respond to the following items on a therapist perceptions of therapeutic alliance scale from 0 (“never”) to 6 (“always”): “Looking back on treatment, I think this child and I worked well together to help with their anxiety” (child–therapist collaboration); “Looking back on treatment, I feel like I liked this child” (therapist–child bond); and “Looking back on treatment, I feel like I liked the child’s caregivers” (therapist–caregiver bond).

### Measures for Study Covariates, Predictors, and Other Included Measures

A number of study covariates, predictors, and other included measures were assessed in the trial. These measures focused on demographic information, youth health and education, adverse childhood experiences (ACEs), experiences with discrimination, mental health stigma, caregiver beliefs about youth anxiety and overprotection, technological literacy, openness to technology-based supports, therapist attitudes and knowledge about youth anxiety and treatment, therapist self-efficacy, and organizational climate of the treatment setting (Table 5).

**Table 5:** Covariates, Predictors, and Other Variables Assessed

Domain	Measure	Informant(s)	Assessment point			
			Baseline	Mid-treatment	Posttreatment	1-Year follow-up
Demographic information	Demographics and background form	C/CS, Y^a^	X			
Youth health and education	Demographics and background form	C, Y^a^	X			
Adverse childhood experiences	CYW ACE-Q; Expanded ACE-Q	C, Y^b^	X	X	X	X
Experiences with discrimination	EDS Short Version	CS, Y^a^	X			
Mental health stigma	PATPSI Stigmatization scale	CS	X			
Beliefs about youth anxiety and overprotection	PABUA Overprotection scale	CS	X	X	X	X
Technological literacy	TECHI	CS, Y^b^, T	X			
Openness to technology-based supports	BATCH-R	CS	X		X	
Therapist attitudes and knowledge						
About evidence-based treatments	EBPAS	T	X^c^^,^^d^			
About exposure therapy	TBES	T	X^c^^,^^d^			
About child anxiety and CBT	Knowledge test	T	X^c^^,^^d^			
Therapist self-efficacy						
CBT/anxiety self-efficacy	TSES-CAY	T	X^c^^,^^d^			
Common factors self-efficacy	TSES-CAY	T	X^c^			
Patient responsivity self-efficacy	TSES-CAY	T	X^c^			
Organizational climate of treatment setting						
Adequacy of resources	TCU ORC scale	T	X^c^			
Organizational climate	TCU ORC scale	T	X^c^			

Note: ACE-Q = Adverse Childhood Experiences Questionnaire; BATCH-R = Beliefs and Attitudes about Technology as a Child Health Resource; C = caregiver report of child; CBT = cognitive behavioral therapy; CS = caregiver self-report; CYW = Center for Youth Wellness; EBPAS = Evidence-Based Practice Attitudes Scale; EDS = Everyday Discrimination Scale; ORC = Organizational Readiness for Change; PABUA = Parental Attitudes, Beliefs, and Understanding of Anxiety; PATPSI = Parental Attitudes Toward Psychological Services Inventory; TBES = Therapist Beliefs about Exposure Scale; TCU = Texas Christian University; TECHI = Technological Ease and Computer Habits Inventory; TSES-CAY = Therapist Self-Efficacy Scale–CBT for Anxiety in Youth; Y = youth self-report.

a Administered to youth ≥11 years.

b Administered to youth ≥13 years.

c Measure administered to therapists prior their onboarding and training.

d Measure administered again to therapists after their training.

#### Demographic Information

Caregivers and youth ≥11 years old provided data on caregiver and youth age, gender, race, ethnicity, nativity (US- or foreign-born), family and household living structure, language(s) spoken, and language(s) preference, among other demographic information. Caregivers also provided information on their child’s grade level, as well as their own highest level of education and literacy comfort. Families were classified as experiencing baseline resource insecurity if the caregiver indicated at baseline that the family experienced any of the following circumstances over the prior 12 months: unhoused or living in a shelter; unable to pay the rent or mortgage on time; the food they purchased did not last, and they did not have money to buy more; there was concern their food would run out before they obtained money to buy more; and the gas or electric company threatened to shut off or refuse gas or electricity to their residence for not paying bills. Administrative records also provided data on family insurance coverage (eg, public, private).

#### Youth Health and Education

Caregivers provided information on the child’s developmental history, medical history, previously diagnosed mental health problems, mental health treatment history, academic performance and attendance, and school accommodations.

#### Adverse Childhood Experiences

The Center for Youth Wellness (CYW) Adverse Childhood Experiences Questionnaire (ACE-Q)^127^ assesses stressful life experiences that can impact child adjustment and development. From a list of 19 specific adversities, caregivers and youth ≥13 years old each reported on the total number of challenging circumstances that participating children and adolescents had endured or encountered. This list included 10 items from the original conceptualization of ACEs (eg, physical, emotion, and sexual abuse; physical and emotional neglect; household dysfunction; living with family members who misuse substances; living with family members with mental illness), as well as a broadened set of 9 additional experiences that can similarly cause prolonged stress that were not included in the original ACEs conceptualization (eg, death of a caregiver, exposure to neighborhood violence, immigration- or deportation-related separation from caregiver, identity-based discrimination, abuse or threats from a romantic partner). Two more items were added to assess whether the child had directly experienced a natural disaster (eg, earthquake, tornado, wildfire, hurricane) or a man-made disaster (eg, terrorist attack, mass shooting, plane crash, industrial fire/explosion, bridge collapse). Respondents reviewed the list of items, tallied the number of these experiences that the child has endured, and reported that total number. Accordingly, the quantity of experienced ACEs was assessed, but information was not collected for the research record that clarifies which specific ACEs were experienced by the child. Per guidelines for the CYW ACE-Q, scores between 1 and 3 indicate “moderate exposure” to ACEs and scores ≥4 are considered high and indicate “considerable exposure” to ACEs.

#### Experiences With Discrimination

The Everyday Discrimination Scale (EDS) Short Version^128^ was used to measure the frequency with which youth and caregivers are subjected to routine experiences of unfair treatment. The EDS Short Version is a brief 5-item adaptation of the original EDS^129^ and has been shown strong reliability and reliability. Respondents indicated how often they experience situations such as being treated with less respect than others, having people act afraid of them, having people act as if they are not smart, and being threatened or harassed. Each item is rated on a frequency scale from 0 (“never”) to 5 (“almost every day”). For responses ≥2, follow-up questions asked respondents to assess what they think is the main reason for these experiences (eg, their ancestry, gender, race, age, religion, weight, sexual orientation, education, or income level). The scale has been used extensively. Higher scores represent more incidences of everyday discrimination compared with lower scores.

#### Mental Health Stigma

The Parental Attitudes Toward Psychological Services Inventory (PATPSI)^130^ stigmatization scale was used to assess the extent to which caregivers are concerned about how others negatively perceive people who have emotional or behavioral health challenges or who seek psychological services. Using a scale ranging from 0 (“strongly disagree”) to 5 (“strongly agree”), respondents rated their level of agreement with 8 items (eg, “I would not want others to know if my child had a psychological or behavioral problem”; “Having been mentally ill carries with it feelings of shame”). The measure has exhibited strong psychometric properties, including a sound factor structure and great reliability and validity.^130^

#### Caregiver Beliefs About Youth Anxiety and Overprotection

The Parental Attitudes, Beliefs, and Understanding of Anxiety (PABUA) Overprotection Scale^131^ is a supported self-report measure that evaluates caregiver attitudes and beliefs about their child’s anxiety and the extent to which they believe they must protect their child from anxiety and distress. Items assess caregiver beliefs about appropriate levels of autonomy granting, whether caregivers believe they should let their anxious child avoid anxiety-provoking situations, and issues of general enmeshment in the caregiver–child relationship. Psychometric research has found the measure and scale to exhibit strong convergent validity, divergent validity, and internal consistency.^131^

#### Technological Literacy

Caregiver and youth technological literacy at baseline was assessed via the Technological Ease and Computer Habits Inventory (TECHI),^132^ which consists of 17 items assessing the extent/frequency of technological usage in everyday life, as well as competency and patience with technology. Items are rated on scales of 0 to 5, and TECHI total score ranges from 0 to 85 (higher scores reflect greater technological usage, competency, and patience). The TECHI has shown strong psychometric properties for assessing technological literacy in the context of technology-based mental health treatment.^132^ To assess baseline therapist technological literacy, therapists also completed the TECHI before training for the trial.

#### Openness to Technology-Based Supports

The Beliefs and Attitudes about Technology as a Child Health Resource (BATCH-R)^133^ is a brief supported self-report that assesses caregiver attitudes (eg, comfort, trust) toward the role of technology in mental health supports and services, parenting information and resources, and professional guidance. The Openness to Technology-Based Mental Health Supports and Treatment scale has caregivers rate from 0 (“strongly disagree”) to 5 (“strongly agree”) their level of agreement with 8 items (eg, “Online computer-based mental health programs can be helpful for treating childhood anxiety”; “I am open to seeking out information online about my child’s health and development”; “I trust the information I receive online about parenting”). The measure has exhibited strong psychometric properties, including a sound factor structure and great reliability and validity.^133^

#### Therapist Attitudes Toward Evidence-Based Treatments

The Evidence-Based Practice Attitudes Scale (EBPAS)^134^ assesses mental health provider attitudes toward evidence-based practices and adopting new interventions. A total of 15 EBPAS items assess the extent to which the therapist would adopt a new practice if it made sense and was used by trusted colleagues (appeal subscale), would adopt a new practice if it was required by their agency (requirements subscale), is open to trying new treatments (openness subscale), or believes research-based interventions are not clinically useful (divergence subscale). Respondents rate their agreement with items on a scale from 0 (“not at all”) to 4 (“to a great extent”), resulting in 4 subscale scores and an EBPAS total score reflecting overall positive disposition toward adopting evidence-based treatments and protocols. Therapists completed the EBPAS before completing their training and again after completing training.

#### Therapist Openness to Exposure Therapy

The Therapist Beliefs about Exposure Scale (TBES)^135^ is a therapist self-report questionnaire that measures negative attitudes about exposure therapy (eg, “Compared to other psychotherapies, exposure therapy leads to higher dropout rates”). Respondents rate their agreement with each of 21 beliefs about exposure therapy on a 5-point scale (0 = “disagree strongly”; 4 = “agree strongly”). Items are summed for a TBES total score (range: 0-84); higher scores reflect more negative views of exposure therapy. Therapists completed the TBES before training for the study and again after training.

#### Therapist Knowledge About Child Anxiety and CBT

An 8-item knowledge test was created to assess therapists’ familiarity with basic research findings about child anxiety (eg, fear is a natural emotion, physical sensations of anxiety cannot harm a child) and its evidence-based treatment (eg, the 3-component model of CBT, the value of exposures). Therapists completed this knowledge test before training for this study and again after training.

#### Therapist Self-Efficacy

Therapists completed the Therapist Self-Efficacy Scale–CBT for Anxiety in Youth (TSES-CAY),^136^ a 16-item survey that measures the extent to which therapists perceive they are capable of competently conducting CBT for youth anxiety. Items from a therapist self-efficacy scale for the treatment of adult depression^137^ were adapted to assess therapist perceptions of their abilities treating anxiety in children and adolescents. TSES-CAY factor analysis has identified a 3-factor structure: CBT/Anxiety-Specific Self-Efficacy, which measures perceived ability to conceptualize client problems using the CBT model, maintain the structure of CBT, teach CBT skills, putting anxious patients in anxiety-provoking situations, and instruct patients to practice skills outside of session; Common Factors Self-Efficacy, which measures perceived ability to build therapeutic alliance, empathize with children/families, etc; and Patient Responsivity Self-Efficacy, which measures perceived ability to adapt to patient/family needs, work collaboratively with patients/families, and address treatment barriers as they arise. Therapists finished all 3 scales of the TSES-CAY before completing their training. After completing their training, therapists again completed the TSES-CAY CBT/Anxiety-Specific Self-Efficacy scale.

#### Organizational Climate of Treatment Setting

The Texas Christian University Organizational Readiness for Change (TCU ORC) scale^138^ was used to assess organizational attributes and motivational factors of the clinical settings of the trial that can impact the treatment implementation success. Before training and onboarding for the trial, therapists, supervisors, administrative directors, and staff members across the clinics participating in the trial completed the TCU ORC subscales that assess adequacy of resources (including offices, staffing, training, equipment, internet, and supervision) and organizational climate (including mission, cohesion, autonomy, communication, stress, and change). The TCU ORC has shown strong psychometric properties. Observed ORC subscale scores can be compared for interpretation against 25th, 50th, and 75th percentile scores reported from national data.^139^

## Discussion

The Kids FACE FEARS trial is poised to offer one of the largest and more inclusive controlled evaluations of treatment for pediatric anxiety. Building on the very strong evidence supporting CBT for the treatment of anxiety, this randomized, multisite, pragmatic effectiveness trial examines 2 CBT delivery formats that vary in therapist intensity, differentially draw on technology, and have shown promise for expanding the reach of care—ie, therapist-led CBT (telehealth, office-based, or hybrid) vs iCBT (with minimal therapist involvement). To evaluate the performance of the treatment comparators under usual care conditions, youth are being screened as part of routine primary or secondary pediatric care, and treatment is being delivered in English and Spanish in usual care settings by usual care providers.

In the context of evolving CBT delivery options and modernized treatment formats that hold great potential to overcome service engagement disparities, the Kids FACE FEARS findings can inform patient-centered decision making and better tailor treatment selections for various groups of underserved youth. Several key features of the trial will advance the generalizability of the findings relative to prior large-scale trials of CBT for youth anxiety. First, the Kids FACE FEARS trial was integrated into usual care in pediatric health settings, whereas many previous large-scale trials have tested treatments in anxiety specialty clinics or laboratory clinic settings, used research therapists paid by the study and who work for the researchers, and/or provided care at no cost to families as part of study participation (eg, ^34^). By integrating study treatment into the normal flow of usual care with usual care providers under usual billing conditions, the Kids FACE FEARS study offers a rare controlled examination of treatment performance under usual care circumstances. Levels of treatment engagement, therapist fidelity, clinical response, and family satisfaction observed in this trial can thus be thought to offer improved generalizability to typical practice settings over prior work.

In addition, eligibility criteria were relaxed relative to previous large-scale trials to better approximate the full range of anxious youth in need of care. By relaxing eligibility criteria in these ways, the trial’s findings can be thought to generalize broadly to “typical” anxious patients presenting in pediatric settings rather than to just selected “pure” diagnostic groups of youth with anxiety disorders who are represented in specialty settings, research laboratories, and narrower efficacy trials.

Further, treatment in the Kids FACE FEARS trial was offered in English and Spanish. Currently, it is estimated that more than 41 million people in the United States speak Spanish, and this number is likely rising. Many single-site trials evaluating treatment for youth anxiety have offered intervention in English and in Spanish,^51^^,^^140^, ^141^, ^142^, ^143^ but large multisite trials to date on youth anxiety treatment have restricted eligibility to English-speaking families. As such, this trial offers a rare large-scale examination of treatment for pediatric anxiety that is more broadly generalizable to the >90% of US households that speak either English or Spanish. Given the expanded language eligibility, along with inclusion of a site that treats an almost entirely Latine population, the Kids FACE FEARS trial will be able to recruit a sample with greater representation of Hispanic and Latine youth and families with foreign-born caregivers. This provides a robust opportunity to study outcomes as they relate to these key underserved and understudied populations.

Importantly, just because technology-based treatment formats can overcome care barriers does not mean in practice they will necessarily expand treatment accessibility beyond populations who already have no difficulty accessing mental health care. Technology-based care overcomes many treatment obstacles, but introduces many new barriers (eg, disparities in technological access and literacy, privacy concerns) and retains many other long-standing treatment barriers, particularly for minoritized youth.^65^^,^^144^^,^^145^ Rhetoric across the literature on technology-based treatments highlights the great promise of technology-based treatment formats for reaching new, previously underserved populations, but there is not much evidence yet that this has been the case at a population level. Technology-based treatment must be focused on more than simply offering more convenient options and alternatives for families who already enjoy access to care.^64^ Given findings such as those from the Kids FACE FEARS trial, the field must redouble its efforts to leverage technology-based treatments to meaningfully broaden the reach of supported care to new and underserved populations.^65^ Technology affords great possibilities, but without focused effort, technology-based care will fall short of its potential.

Findings from the Kids FACE FEARS trial will need to be interpreted in the context of several study limitations. The first set of limitations pertains to sample eligibility and size. Children younger than age 7 were excluded to focus on the age ranges of youth most seen in practice settings. Parenting-focused CBT has been found to be very effective for early child anxiety,^31^ and research supports both telehealth and online computerized formats for delivering care.^51^^,^^56^ Research will be needed to consider the relative utility of these 2 formats for treating anxiety at earlier developmental stages than included in this trial. In addition, in this study, the treatment comparators were offered in English and in Spanish. Although an estimated 92% of the US population speaks at least 1 of these languages, there are many other languages spoken in US households. To more comprehensively address linguistic disparities in care, future trials should examine the effectiveness of CBT formats in other languages beyond English and Spanish. Moreover, power analysis for the present trial was based on the primary comparative effectiveness aims of the trial. Although sensitivity analysis determined the sample size was also sufficient to detect moderation effects that are small in magnitude, it is possible the large number of secondary variables and constructs being measured could introduce statistical power concerns.

A second set of limitations pertains to what was not assessed in the Kids FACE FEARS trial. Funding considerations did not allow for longer-term follow-up beyond 1 year. As such, the findings of the trial will not be able to speak to the longer-term endurance of treatment gains into young adulthood. In addition, the streamlined assessment protocol focused on patient-centered outcomes that minimized burden to families and staff. As such, structured diagnostic interviews (which typically take several hours to complete, are rarely feasible in frontline practice settings, and rarely produce diagnoses that align with diagnoses assigned in usual care settings^90^) were not included in this trial. Although the PARS was conducted by masked IEs, the Kids FACE FEARS trial will not be able to speak to findings as they relate to specific *DSM-5-TR* anxiety diagnoses. Also, studying the treatment comparators as delivered by natural providers (ie, not research therapists) under typical care circumstances (ie, not in laboratory clinics) did not allow for recording of sessions or support calls. Accordingly, data on independently rated treatment fidelity and alliance will not be available, although caregiver, youth, and therapist perspectives of these variables were collected. Moreover, Kids FACE FEARS assessments did not ask about youth sexual orientation. The prevalence of anxiety has been increasing among sexual minority teens,^18^ a population that experiences unique barriers to care. Data from this trial will not be able to analyze sexual minority status as it relates to treatment responses.

A third set of limitations pertains to where the Kids FACE FEARS trial was conducted. Specifically, the study was conducted at high-volume, urban pediatric health care sites affiliated with 4 major medical centers that predominantly serve youth from urban and suburban communities. Geographic disparities in mental health care availability and quality leave rural youth critically underserved.^146^ Telehealth and computerized options for CBT delivery hold great promise for rural youth,^147^^,^^148^ but findings from this trial cannot speak to this important population. Furthermore, the Kids FACE FEARS trial focused on identifying anxious youth and providing services in pediatric health care settings, but there are many other sectors where youth receive anxiety services and supports, including schools and community mental health centers.^45^^,^^149^, ^150^, ^151^ Pediatric health settings offer a key opportunity to reach large numbers of youth, but similar research must evaluate the performance of the treatment comparators in other key service sectors to collectively reach an even broader proportion of anxious youth.

A fourth set of limitations is related to what specific treatments were studied for the CBT comparators. The Cool Kids suite of treatments has received strong support, but there are many other exceptional CBT protocols for youth anxiety (eg,^36^^,^^52^, ^53^, ^54^^,^^57^, ^58^, ^59^^,^^152^, ^153^, ^154^, ^155^) that could have been selected. Further, the Kids FACE FEARS trial examined anxiety-specific treatment, but research also shows support for transdiagnostic treatment approaches that can flexibly treat a range of youth problems.^156^^,^^157^ Moreover, the online program selected for the iCBT comparator is a structured, modularized program that was not designed as a mobile intervention. As such, it does not include automated and personalized capabilities (ie, “smart” features), incorporate wearable technologies or ecological momentary assessments, use artificial intelligence or machine learning algorithms, or respond in a “just-in-time” manner to shifting patient states in moments of particular need. Exciting new digital mental health tools are now beginning to leverage these “smarter” features and functionalities,^57^^,^^158^, ^159^, ^160^ and thus evolving digital mental health innovations for pediatric anxiety may come to outperform the findings of the Kids FACE FEARS trial.

On a final note, the Kids FACE FEARS trial was conducted across a time of unprecedented challenges and extraordinary hardships, strains, and demands for families. At the start of the trial, the COVID-19 pandemic ushered in a period of tremendous threat, loss, disruption, and social isolation, and the burdens of the pandemic were not shared equally across the population. This all unfolded against a backdrop of increased division and social unrest, widening disparities, shifting policies for telehealth practice, and the politicization of public health. Such factors may have collectively impacted the nature of the anxiety experienced by the children presenting for care in the trial, as well as family treatment engagement, youth anxiety response, and levels of study attrition. The generalizability and interpretation of all research is limited, to some extent, by the times in which the work is conducted, and the Kids FACE FEARS trial is no different. It is possible that the nature of pediatric anxiety and its response to the treatment comparators may fare differently during less overwhelming times for families.

In conclusion, the number of underserved youth with impairing anxiety is staggering and rising. Building on the strong evidence supporting CBT for the treatment of pediatric anxiety, the Kids FACE FEARS trial was designed to address noted gaps in understanding the effectiveness of modernized CBT delivery formats that vary in therapist intensity and technological involvement. Findings from this multisite comparative effectiveness trial will be able to attest to the extent to which such modernized CBT formats can play meaningful roles in extending the reach of care for youth who experience anxiety in usual care settings and perhaps produce population-level impacts.

## CRediT authorship contribution statement

**Jonathan S. Comer:** Writing – review & editing, Writing – original draft, Visualization, Supervision, Project administration, Methodology, Investigation, Funding acquisition, Formal analysis, Data curation, Conceptualization. **Donna B. Pincus:** Writing – review & editing, Writing – original draft, Supervision, Project administration, Investigation, Funding acquisition, Conceptualization. **Molly C. Adrian:** Writing – review & editing, Supervision, Project administration, Investigation. **Gary McCreary:** Writing – review & editing, Investigation, Conceptualization. **Leslie Miller:** Writing – review & editing, Supervision, Project administration, Investigation. **Tomas Munarriz:** Conceptualization, Investigation, Writing – review & editing. **Kathleen Myers:** Writing – review & editing, Supervision, Funding acquisition, Conceptualization. **Karen Xiomara Pierre-Louis:** Writing – review & editing, Investigation, Conceptualization. **Rheanna Platt:** Writing – review & editing, Supervision, Project administration, Investigation. **Melissa K. Ripley:** Writing – review & editing, Investigation, Conceptualization. **Andrea E. Spencer:** Writing – review & editing, Supervision, Project administration, Investigation, Funding acquisition, Conceptualization. **Haniya Saleem Syeda:** Writing – review & editing, Project administration, Methodology, Investigation. **Margarita Alegría:** Writing – review & editing, Investigation. **Amelia Brandt:** Writing – review & editing, Supervision, Project administration. **Carolina Costa:** Writing – review & editing, Project administration, Data curation. **Lindsay Cooper:** Writing – review & editing, Supervision, Project administration. **Stefany Coxe:** Writing – review & editing, Methodology, Formal analysis, Data curation. **Annie W. Dantowitz:** Writing – review & editing, Supervision, Project administration. **Anthony Steven Dick:** Writing – review & editing, Visualization, Formal analysis, Data curation. **Alyssa M. Farley:** Writing – review & editing, Supervision, Project administration, Formal analysis. **Jami M. Furr:** Writing – review & editing, Supervision, Project administration. **Alex E. Keller:** Writing – review & editing, Supervision, Project administration. **Julia A. Lejeune:** Writing – review & editing, Project administration, Data curation. **Lauren F. McLellan:** Writing – review & editing, Software, Resources, Project administration. **Dana L. McMakin:** Writing – review & editing, Supervision, Project administration, Investigation, Funding acquisition, Conceptualization. **Rachel A. Merson:** Writing – review & editing, Supervision, Project administration. **Ricardo F. Muñoz:** Writing – review & editing, Investigation. **Ronald M. Rapee:** Writing – review & editing, Software, Resources, Investigation. **Kendra L. Read:** Writing – review & editing, Supervision, Project administration. **Sara Rivero-Conil:** Writing – review & editing, Supervision, Project administration. **Bridget Poznanski:** Writing – review & editing, Formal analysis, Data curation. **Michelin Jane Janvier:** Writing – review & editing, Project administration. **Hanan N. Salem:** Writing – review & editing, Visualization, Project administration, Methodology, Investigation, Data curation, Conceptualization. **Philip Shumway:** Writing – review & editing, Project administration, Data curation. **Jennifer Sikov:** Writing – review & editing, Project administration, Data curation. **Michelle V. Porche:** Writing – review & editing, Software, Investigation, Conceptualization. **Lisa R. Fortuna:** Writing – review & editing, Project administration, Investigation, Conceptualization.
